# *catena*-Poly[[dichloridocobalt(II)]-μ-1,3-di-4-pyridylpropane-κ^2^ *N*:*N*′]

**DOI:** 10.1107/S1600536808020862

**Published:** 2008-07-12

**Authors:** Wei Xu, Jian-Li Lin

**Affiliations:** a State Key Laboratory Base of Novel Functional Materials and Preparation Science, Faculty of Materials Science and Chemical Engineering, Ningbo University, Ningbo 315211, People’s Republic of China

## Abstract

In the title compound, [CoCl_2_(C_13_H_14_N_2_)]_*n*_, 1,3-bis­(4-pyrid­yl)propane (bpp) ligands bridge four-coordinate Co atoms, generating an extended one-dimensional zigzag chain. Both the Co and two Cl atoms in the tetrahedral coordination polyhedron lie on a mirror plane, while the bbp ligand is bis­ected through the central C atom in the chain by a second mirror plane. There are some π–π stacking inter­ations in the crystal structure, with inter­planar distances of 3.449 Å, which are responsible for the supra­molecular assembly.

## Related literature

For related literature, see: Batten *et al.* (1999 ▶); Chen *et al.* (2004 ▶); Grosshans *et al.* (2004 ▶); Lee *et al.* (2004 ▶); Maji *et al.* (2005 ▶); Niu *et al.* (2003 ▶); Paz & Klinowski (2004 ▶); Carlucci *et al.* (1997 ▶); Pan *et al.* (2001 ▶).
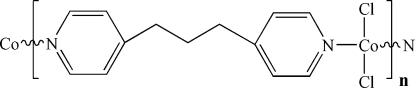


## Experimental

### 

#### Crystal data

[CoCl_2_(C_13_H_14_N_2_)]
*M* *_r_* = 328.09
Monoclinic, 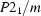

*a* = 5.1899 (10) Å
*b* = 12.989 (3) Å
*c* = 10.490 (2) Å
β = 93.58 (3)°
*V* = 705.8 (3) Å^3^
*Z* = 2
Mo *K*α radiation
μ = 1.58 mm^−1^
*T* = 295 (2) K
0.36 × 0.25 × 0.13 mm

#### Data collection

Rigaku R-AXIS RAPID diffractometer
Absorption correction: multi-scan (*ABSCOR*; Higashi, 1995 ▶) *T*_min_ = 0.628, *T*_max_ = 0.813
6891 measured reflections
1678 independent reflections
1307 reflections with *I* > 2σ(*I*)
*R*_int_ = 0.057

#### Refinement

*R*[*F* ^2^ > 2σ(*F* ^2^)] = 0.043
*wR*(*F* ^2^) = 0.098
*S* = 1.03
1678 reflections
88 parameters
H-atom parameters constrained
Δρ_max_ = 0.56 e Å^−3^
Δρ_min_ = −0.32 e Å^−3^

### 

Data collection: *RAPID-AUTO* (Rigaku, 1998 ▶); cell refinement: *RAPID-AUTO*; data reduction: *CrystalStructure* (Rigaku/MSC, 2004 ▶); program(s) used to solve structure: *SHELXS97* (Sheldrick, 2008 ▶); program(s) used to refine structure: *SHELXL97* (Sheldrick, 2008 ▶); molecular graphics: *ORTEPII* (Johnson, 1976 ▶); software used to prepare material for publication: *SHELXL97*.

## Supplementary Material

Crystal structure: contains datablocks global, I. DOI: 10.1107/S1600536808020862/bg2192sup1.cif

Structure factors: contains datablocks I. DOI: 10.1107/S1600536808020862/bg2192Isup2.hkl

Additional supplementary materials:  crystallographic information; 3D view; checkCIF report

## Figures and Tables

**Table 1:** Selected bond lengths (Å)

Co1—N1	2.034 (2)
Co1—Cl1	2.2400 (14)
Co1—Cl2	2.2539 (14)

**Fig. 1.**
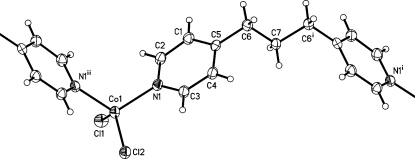
ORTEP view of the title compound. The dispalcement ellipsoids are drawn at 40% probability level.

**Fig. 2.**
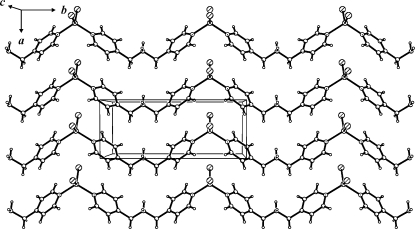
The crystal packing of the title complex, view pallel to (001).

## supplementary crystallographic information

### Comment

Transition metal complexes with the flexible ligand 1,3-bis(4-pyridyl)propane (bpp) have been investigated extensively (Pan, *et al.*, 2001; Batten, *et al.*, 1999; Carlucci, *et al.*, 1997; Lee, *et al.*, 2004), and some of these compounds have potential application in nonlinear optical (NLO), magnetic, gas adsorption and microporous materials (Maji, *et al.*, 2005; Niu, *et al.*, 2003; Paz, *et al.*, 2004). Our interest in transition metal-bpp complexes prompted us to report a new bpp-containing complex, [Co(bpp)Cl_2_]_n_, (I), obtained by self-assembly from CoCl_2_ and bpp in DMF solution. It is isostructural with the previously reported complex [Zn(bpp)Cl_2_]_n_ (Chen, *et al.*, 2004).

In the title compound, the Co atom is coordinated by two N atoms of two bpp ligands and two Cl anions, forming a distorted tetrahedron (Figure 1 and Table 1.) Both the cation and two chlorine atoms in the coordination polyhedra lie on a mirror plane, while the bbp ligand is bisected through the central carbon in the chain by a second mirror. The angles around Co(II) ions span the range 104.2° to 125. 7°. The Co—N bond distance is 2.034 (2) Å, and the Co—Cl distances are 2.240 (1) and 2.254 (1) Å, similar to those found in other related structures (Grosshans, *et al.*, 2004). The bpp ligand is in a TT conformation, with a dihedral angle between two pyridine rings of 64.9°, and a C5—C6—C7—C6^i^ (i = -*x*, 1.5 - *y*, *z*) torsion angle of 176.0°. Each bpp ligand bridges two cobalt(II) ions together *via* nitrogen atoms to form one-dimensional zigzag chains; there are π-π interations between pyridine rings, which are arranged in a face-to-face fashion with interplanar distances of 3.449 Å.

### Experimental

Addition of 1,3-bis(4-pyridyl)propane (bpp) (0.198 g, 1.0 mmol) to a stirred DMF solution (30 ml) of CoCl_2_.6H_2_O (0.207 g 1.0 mmol) yielded a purple precipitate, which was refluxed for 30 min at 423 K followed by filtration after cooling. The resulting blue filtrate was maintained at room temperature; slow evaporation afforded a small amount of purple platelet cystals 15 days later (yield: 42% based on the initial CoCl_2_.6H_2_O input).

### Refinement

All H atoms werer located theoretically and refined as riding atoms, with C—H distances in the range 0.93–0.97 Å and *U*_iso_(H) = 1.2 *U*_eq_(C).

### Crystal data

**Table:**

[CoCl_2_(C_13_H_14_N_2_)]	*F*_000_ = 334
*M**_r_* = 328.09	*D*_x_ = 1.544 Mg m^−^^3^
Monoclinic, *P*2_1_/*m*	Mo *K*α radiation λ = 0.71073 Å
Hall symbol: -P 2yb	Cell parameters from 4916 reflections
*a* = 5.1899 (10) Å	θ = 3.1–27.5º
*b* = 12.989 (3) Å	µ = 1.58 mm^−^^1^
*c* = 10.490 (2) Å	*T* = 295 (2) K
β = 93.58 (3)º	Palte, purple
*V* = 705.8 (3) Å^3^	0.36 × 0.25 × 0.13 mm
*Z* = 2	

### Data collection

**Table:**

Rigaku R-AXIS RAPID diffractometer	1678 independent reflections
Radiation source: fine-focus sealed tube	1307 reflections with *I* > 2σ(*I*)
Monochromator: graphite	*R*_int_ = 0.057
Detector resolution: 0 pixels mm^-1^	θ_max_ = 27.5º
*T* = 295(2) K	θ_min_ = 3.1º
ω scans	*h* = −6→6
Absorption correction: multi-scan(ABSCOR; Higashi, 1995)	*k* = −16→16
*T*_min_ = 0.628, *T*_max_ = 0.813	*l* = −13→13
6891 measured reflections	

### Refinement

**Table:**

Refinement on *F*^2^	Secondary atom site location: difference Fourier map
Least-squares matrix: full	Hydrogen site location: inferred from neighbouring sites
*R*[*F*^2^ > 2σ(*F*^2^)] = 0.043	H-atom parameters constrained
*wR*(*F*^2^) = 0.098	*w* = 1/[σ^2^(*F*_o_^2^) + (0.0331*P*)^2^ + 0.5699*P*] where *P* = (*F*_o_^2^ + 2*F*_c_^2^)/3
*S* = 1.03	(Δ/σ)_max_ < 0.001
1678 reflections	Δρ_max_ = 0.56 e Å^−^^3^
88 parameters	Δρ_min_ = −0.32 e Å^−^^3^
Primary atom site location: structure-invariant direct methods	Extinction correction: none

### Special details

**Table:**

Geometry. All e.s.d.'s (except the e.s.d. in the dihedral angle between two l.s. planes) are estimated using the full covariance matrix. The cell e.s.d.'s are taken into account individually in the estimation of e.s.d.'s in distances, angles and torsion angles; correlations between e.s.d.'s in cell parameters are only used when they are defined by crystal symmetry. An approximate (isotropic) treatment of cell e.s.d.'s is used for estimating e.s.d.'s involving l.s. planes.
Refinement. Refinement of *F*^2^ against ALL reflections. The weighted *R*-factor *wR* and goodness of fit *S* are based on *F*^2^, conventional *R*-factors *R* are based on *F*, with *F* set to zero for negative *F*^2^. The threshold expression of *F*^2^ > σ(*F*^2^) is used only for calculating *R*-factors(gt) *etc*. and is not relevant to the choice of reflections for refinement. *R*-factors based on *F*^2^ are statistically about twice as large as those based on *F*, and *R*- factors based on ALL data will be even larger.

### Fractional atomic coordinates and isotropic or equivalent isotropic displacement parameters (Å^2^)

**Table:**

	*x*	*y*	*z*	*U*_iso_*/*U*_eq_	
Co1	0.45224 (11)	0.2500	0.72216 (5)	0.0469 (2)	
Cl1	0.5449 (2)	0.2500	0.51643 (11)	0.0644 (3)	
Cl2	0.7429 (2)	0.2500	0.89105 (11)	0.0563 (3)	
N1	0.2438 (5)	0.38105 (17)	0.7418 (2)	0.0456 (5)	
C1	−0.1030 (6)	0.4919 (2)	0.6704 (3)	0.0541 (8)	
H1A	−0.2369	0.5056	0.6097	0.065*	
C2	0.0502 (6)	0.4066 (2)	0.6576 (3)	0.0543 (8)	
H2A	0.0175	0.3645	0.5867	0.065*	
C3	0.2864 (6)	0.4443 (2)	0.8422 (3)	0.0515 (7)	
H3A	0.4195	0.4283	0.9025	0.062*	
C4	0.1436 (6)	0.5310 (2)	0.8601 (3)	0.0528 (7)	
H4A	0.1821	0.5727	0.9309	0.063*	
C5	−0.0580 (6)	0.5572 (2)	0.7735 (3)	0.0463 (7)	
C6	−0.2140 (6)	0.6529 (2)	0.7913 (3)	0.0523 (7)	
H6A	−0.2666	0.6554	0.8784	0.063*	
H6B	−0.3688	0.6506	0.7346	0.063*	
C7	−0.0613 (8)	0.7500	0.7639 (4)	0.0476 (9)	
H7A	−0.0195	0.7500	0.6750	0.057*	
H7B	0.0993	0.7500	0.8163	0.057*	

### Atomic displacement parameters (Å^2^)

**Table:**

	*U*^11^	*U*^22^	*U*^33^	*U*^12^	*U*^13^	*U*^23^
Co1	0.0569 (4)	0.0384 (3)	0.0452 (3)	0.000	0.0009 (3)	0.000
Cl1	0.0673 (7)	0.0813 (8)	0.0442 (6)	0.000	0.0004 (5)	0.000
Cl2	0.0634 (7)	0.0500 (6)	0.0539 (6)	0.000	−0.0076 (5)	0.000
N1	0.0506 (13)	0.0385 (11)	0.0474 (14)	−0.0043 (11)	0.0002 (11)	0.0027 (10)
C1	0.0570 (17)	0.0463 (16)	0.0573 (19)	−0.0023 (15)	−0.0108 (15)	0.0018 (13)
C2	0.0651 (19)	0.0453 (16)	0.0514 (18)	−0.0055 (15)	−0.0057 (15)	−0.0044 (13)
C3	0.0586 (18)	0.0419 (15)	0.0528 (18)	0.0007 (14)	−0.0070 (14)	−0.0038 (12)
C4	0.0604 (18)	0.0430 (15)	0.0541 (18)	−0.0034 (15)	−0.0029 (14)	−0.0070 (13)
C5	0.0475 (15)	0.0373 (14)	0.0545 (17)	−0.0088 (12)	0.0053 (13)	0.0040 (12)
C6	0.0515 (16)	0.0409 (15)	0.065 (2)	−0.0007 (14)	0.0092 (15)	0.0043 (13)
C7	0.050 (2)	0.0363 (19)	0.057 (3)	0.000	0.0068 (19)	0.000

### Geometric parameters (Å, °)

**Table:**

Co1—N1^i^	2.034 (2)	C3—H3A	0.9300
Co1—N1	2.034 (2)	C4—C5	1.385 (4)
Co1—Cl1	2.2400 (14)	C4—H4A	0.9300
Co1—Cl2	2.2539 (14)	C5—C6	1.501 (4)
N1—C2	1.338 (4)	C6—C7	1.526 (4)
N1—C3	1.343 (4)	C6—H6A	0.9700
C1—C2	1.375 (4)	C6—H6B	0.9700
C1—C5	1.383 (4)	C7—C6^ii^	1.526 (4)
C1—H1A	0.9300	C7—H7A	0.9700
C2—H2A	0.9300	C7—H7B	0.9700
C3—C4	1.368 (4)		
			
N1^i^—Co1—N1	113.61 (13)	C3—C4—C5	120.4 (3)
N1^i^—Co1—Cl1	104.19 (7)	C3—C4—H4A	119.8
N1—Co1—Cl1	104.19 (7)	C5—C4—H4A	119.8
N1^i^—Co1—Cl2	104.73 (7)	C1—C5—C4	116.5 (3)
N1—Co1—Cl2	104.73 (7)	C1—C5—C6	122.6 (3)
Cl1—Co1—Cl2	125.73 (5)	C4—C5—C6	120.9 (3)
C2—N1—C3	116.5 (3)	C5—C6—C7	111.7 (3)
C2—N1—Co1	121.5 (2)	C5—C6—H6A	109.3
C3—N1—Co1	121.9 (2)	C7—C6—H6A	109.3
C2—C1—C5	120.0 (3)	C5—C6—H6B	109.3
C2—C1—H1A	120.0	C7—C6—H6B	109.3
C5—C1—H1A	120.0	H6A—C6—H6B	107.9
N1—C2—C1	123.4 (3)	C6—C7—C6^ii^	111.4 (3)
N1—C2—H2A	118.3	C6—C7—H7A	109.3
C1—C2—H2A	118.3	C6^ii^—C7—H7A	109.3
N1—C3—C4	123.2 (3)	C6—C7—H7B	109.3
N1—C3—H3A	118.4	C6^ii^—C7—H7B	109.3
C4—C3—H3A	118.4	H7A—C7—H7B	108.0
			
C5—C6—C7—C6^ii^	−176.0 (2)		

Symmetry codes: (i) *x*, −*y*+1/2, *z*; (ii) *x*, −*y*+3/2, *z*.

## References

[bb1] Batten, S. R., Jeffey, J. C. & Ward, M. D. (1999). *Inorg. Chim. Acta*, **292**, 231–237.

[bb2] Carlucci, L., Ciani, G. W., Gudenberg, D. & Proserpio, D. M. (1997). *Inorg. Chem.* **36**, 3812–3813.

[bb3] Chen, Y.-B., Kang, Y., Qin, Y.-Y., Li, Z.-J., Cheng, J.-K., Hu, R.-F., Wen, Y.-H. & Yao, Y.-G. (2004). *Acta Cryst.* C**60**, m168–m169. 10.1107/S0108270104003592 15071206

[bb4] Grosshans, P., Jouaiti, A., Bulach, V., Planeix, J. M., Hosseini, M. W. & Kyritsakas, N. (2004). *Eur. J. Inorg. Chem.* pp. 453–458.

[bb5] Higashi, T. (1995). *ABSCOR* Rigaku Corporation, Tokyo, Japan.

[bb6] Johnson, C. K. (1976). *ORTEPII* Report ORNL-5138. Oak Ridge National Laboratory, Tennessee, USA.

[bb7] Lee, T. W., Lau, J. P. K. & Wong, W. T. (2004). *Polyhedron*, **23**, 999–1002.

[bb8] Maji, T. K., Mostafa, G., Matsuda, R. & Kitagawa, S. (2005). *J. Am. Chem. Soc.* **127**, 17152–17153. 10.1021/ja0561439 16332040

[bb9] Niu, Y. Y., Song, Y. L., Hou, H. W. & Zhu, Y. (2003). *Inorg. Chim. Acta*, **355**, 151–156.

[bb10] Pan, L., Woodlock, W. B., Wang, X. T., Lam, K. C. & Rheingold, A. L. (2001). *Chem. Commun.* pp. 1762–1763. 10.1039/b104074j 12240303

[bb11] Paz, F. A. A. & Klinowski, J. (2004). *Inorg. Chem.* **43**, 3882–3893. 10.1021/ic049523o 15206869

[bb12] Rigaku (1998). *RAPID-AUTO* Rigaku Corporation, Tokyo, Japan.

[bb13] Rigaku/MSC (2004). *CrystalStructure* Rigaku/MSC Inc., The Woodlands, Texas, USA.

[bb14] Sheldrick, G. M. (2008). *Acta Cryst.* A**64**, 112–122. 10.1107/S0108767307043930 18156677

